# Incorporation of Herbal Essential Oils in Chicken Sausages and Their Effects on Microbial Stability and Product Quality

**DOI:** 10.3390/foods14101756

**Published:** 2025-05-15

**Authors:** Janeth Proaño, María Paula Urresta, Lucía Toledo, Daniel Polo, Pablo Moncayo, Wilson Vásquez-Castillo

**Affiliations:** 1 Ingeniería Agroindustrial, Universidad de Las Américas (UDLA), Quito 170124, Ecuador; 2 Facultad de Ingenierías y Ciencias Aplicadas, Universidad de Las Américas (UDLA), Quito 170124, Ecuador

**Keywords:** food industry, preservatives, shelf life, natural additives, microorganisms, additives

## Abstract

The global food industry primarily relies on synthetic preservatives and additives to extend shelf life and enhance product quality. The growing demand for clean-label meat products has prompted interest in natural preservatives. This study aimed to evaluate the antimicrobial effect of thyme (*Thymus vulgaris*), oregano (*Origanum vulgare*) and clove (*Syzygium aromaticum*) essential oils’ physicochemical and organoleptic characteristics in chicken sausages. In vitro, assays assessed antimicrobial activity at concentrations ranging from 40 µg kg^−1^ to 600 µg kg^−1^, while in vivo trials evaluated the effects of encapsulated and non-encapsulated essential oils during 30 days of refrigerated storage. The treatments differed in terms of essential oil, dose, combination and method of application. The results showed that the essential oils, at different doses and in combination, controlled the growth (28%) and presence of the evaluated microorganisms (*C. perfringens*, *S. aureus*, *E. coli* and *Salmonella*) in the chicken sausages. Moreover, the non-encapsulated application of essential oils demonstrated greater antimicrobial effectiveness compared to the encapsulated form. Overall, the results support the potential of these plant essential oils as safe, effective alternatives to synthetic preservatives in chicken sausage, without negatively affecting nutritional or sensory attributes.

## 1. Introduction

The global food industry mostly uses synthetic preservatives and additives in order to extend product shelf life and ensure quality while maintaining the food’s organoleptic, physical and chemical properties [1], as well as preventing the growth of microorganisms that can affect people’s health. Nevertheless, growing evidence links these synthetic preservatives to antimicrobial resistance, toxicity and even potential carcinogenicity [2]. For instance, synthetic food additives used in formulations have been associated with an increased risk of cancer. These effects can impact various organs and tissues [3]. In this context, the Pan American Health Organization (PAHO) and the World Health Organization (WHO) [4] report that several components of red and processed meat are linked to increased cancer risks. This finding is supported by other authors [5,6]. The WHO has estimated that up to 30% of cancer cases could be prevented if people adopt a nutritionally balanced and health-promoting diet [7].

Over the years, countless studies have shown that people’s nutrition is fundamental to health, as it prevents chronic diseases associated with food consumption [7]. As a result, consumers increasingly favour foods that go beyond meeting basic nutritional needs. They look for products that contribute to disease prevention and promote both physical and mental well-being [8].

Due to the health concerns associated with synthetic additives, the food industry is seeking natural alternatives to replace them. In this context, certain plant essential oils exhibit antibacterial, antifungal, insecticidal and antiviral properties, primarily due to active compounds such as terpenes and phenylpropanoids [9,10].

In the manufacture of processed chicken sausages, it is necessary to use preservatives, which include nitrates, erythorbates, benzoates and antioxidants such as butyl hydroxyanisole and butyl hydroxytoluene, permitted by the Ecuadorian regulation INEN 1338:2016 but which, if consumed in high quantities, can affect the health of the consumer [11].

Meanwhile, the global trend in meat consumption is increasing, with mortadella, hams and sausages being the most prominent [12]. Sausages provide an affordable source of protein, which is particularly important for socioeconomic groups with limited financial resources [8]. In addition, many sausages are produced using meat by-products (such as trimmings, fat, viscera, skin and blood), thereby reducing waste after the primary meat cuts have been removed from the carcass [12]. Considering that 25.5% of Ecuador’s population lives in poverty and 9.5% in extreme poverty [13], sausages represent a viable protein source for these vulnerable sectors of the population [8]. In Ecuador, mortadella and sausages account for 75% of the country’s processed meat production, followed by chorizo (14%), ham (5%) and other types of sausages (6%) [14]. The average per capita consumption of sausages in Ecuador is 4.1 kg, which represents 16% of food and beverages in the Ecuadorian basic food basket [15].

The application of essential oils as biopreservatives in food can inhibit pathogens due to their broad-spectrum antimicrobial activity, which is attributed to the presence of secondary metabolites, aromatic compounds and volatile constituents [16]. In recent years, the use of biopreservatives has been studied in different food matrices [17] as a possible replacement for synthetic preservatives in sausages.

Bacteria such as *Staphylococcus aureus*, *Escherichia coli*, *Salmonella* spp., mesophilic aerobes and *Clostridium perfringens* (NTE INEN 1338:2016) could proliferate in sausages when stored under refrigeration for a period of 30 days. The essential oils of oregano (*Origanum vulgaris*), thyme (*Thymus vulgaris*) and clove (*Syzygium aromaticum*) are known to have selective antibacterial properties [18]. Oregano essential oil possesses a bioactive compound consisting of terpenes, mainly mono- and sesquiterpenes [19]. The main terpenes identified in oregano essential oils are carvacrol, thymol, γ-terpinene and p-cymene, followed by terpinen-4-ol, linalool, β-myrcene, trans-sabinene hydrate and β-caryophyllene [20]. Carvacrol and thymol in the mixture have a positive effect on total inhibition against *Pseudomonas aeruginosa*, *E. coli*, *Listeria monocytogenes*, *S. aureus* and *Salmonella typhimurium* [21].

The 4% essential oil of oregano applied to refrigerated tilapia fillets controlled microorganisms such as *S. aureus*, mesophilic aerobes, facultative psychrophilic anaerobes and *E. coli* and ensured the absence of *Salmonella* [22]. Studies carried out by Carhuallanqui Pérez et al. [9] showed that oregano essential oil had better antibacterial activity on Gram-positive strains. However, the tested bacteria—*Bacillus subtilis*, *Staphylococcus aureus* and *Staphylococcus saprohyticus*—were more sensitive to oregano essential oil than to the antibiotic ampicillin. Research carried out by Ozogul et al. [23] indicated the efficiency of rosemary and thyme essential oil for the control of mesophilic aerobes in fish fillets. In addition, the oils maintained the physicochemical properties of the product, including pH, shelf life and organoleptic characteristics [22].

Thyme essential oil inhibited *E. coli* and *S. aureus* at a concentration of 9.17 mg/mL in meat sausages and partially counteracted mesophilic bacteria [24]; similarly, it prevented the proliferation of *Clostridium perfringens* [25]. Meanwhile, essential oils of clove, thyme, *Cassia* sp. and basil placed in sausages were well accepted in sensory analyses [26].

Given the above, the aim of the study was to evaluate the antimicrobial effect of thyme (*Thymus vulgaris*), oregano (*Origanum vulgare*) and clove (*Syzygium aromaticum*) essential oils’ physicochemical and organoleptic characteristics in chicken sausage in order to reduce or replace synthetic preservatives.

## 2. Materials and Methods

The study was conducted in three phases: (1) an in vitro antimicrobial evaluation of the essential oils of the three plant species, (2) an antimicrobial evaluation of the essential oils in chicken sausages and (3) an organoleptic evaluation of the chicken sausages.

### 2.1. In Vitro Antimicrobial Evaluation of Essential Oils

In order to determine the antibacterial effect of oregano, thyme and clove essential oils, the in vitro disc diffusion method known as the Kirby-Bauer test was used [27], which is a technique widely used in microbiology to determine microorganisms’ antimicrobial susceptibility [28]. This method involves placing sterilised filter paper discs on the surface of the Petri dish with the agar, on which the bacteria to be tested were previously inoculated [29].

Prior to inoculation, *Escherichia coli*, *Salmonella*, *Staphylococcus aureus*, mesophilic aerobes and *Clostridium perfingens* were placed in tubes with 10 mL of peptone water and incubated in a growth chamber (Model Memmert IN-500, Caracas, Venezuela, 2013) at 37 °C for 24–36 h. After this, 1 mL of the solution (bacteria and peptone) was removed and inoculated into glass tubes containing 9 mL of saline. In these tubes, the turbidity was measured and standardised to 0.5 MC Farland (Model DINKO, Barcelona, Spain, 2017) units (the sample of each bacterium corresponding to 1.5 × 10^8^ cfu/mL).

In standardised tubes containing the bacterial saline solution, 0.1 mL of each bacterium was inoculated in triplicate into Petri dishes containing agar according to the following specifications. *E. coli* and *Salmonella* were grown on EMB agar, *Staphylococcus aureus* on Mannitol Salt agar, mesophilic aerobes on PCA agar and *Clostridium perfingens* on SPS agar. The 4 discs were then placed in the Petri dishes containing the agar with the bacteria. The amounts of the essential oils (established treatments) listed in Table 1 were added to the sterile discs and placed in the incubator at 37 °C for 24–48 h. *Clostridium perfingens* was placed in the anaerobic chamber and incubated. At the end of the incubation period, the diameter of the inhibition zone (DIZ) around the disc was measured with a ruler. A larger DIZ was interpreted as indicating greater antibacterial effectiveness. The sensitivity scale recommended by Picazo [30] was used, with the following categories: null sensitivity [(−) < 8 mm]; sensitive [(+) > 8 mm ≤ 14 mm]; very sensitive [(++) > 14–20 mm]; and extremely sensitive [(+++) > 20 mm].

### 2.2. Determination of the Antimicrobial Effect of Essential Oils in Chicken Sausages

The amount of essential oil (%) was determined on the basis of the minimum inhibitory concentrations obtained from the in vitro study, which were added (without encapsulation (E−) or with encapsulation (E+)) to the chicken sausage according to the details of the treatments outlined in Table 1. The dose of clove essential oil was constant across all treatments since studies have shown that this amount of biopreservative controls the growth of *Clostridium perfringens* due to the presence of eugenol [31].

### 2.3. Encapsulation of Essential Oils

The encapsulates were prepared following the protocol described by Delshadi et al. [32]. The dosages were established for samples of 300 g of meat product (chicken sausages), i.e., a 0.2% alginate solution with respect to the weight of the sample and 50 mL of distilled water. A CaCl_2_ solution was prepared at 2% by weight of distilled water. Treatments were individually placed in different alginate solutions. Using a 5 mL plastic syringe, the solutions containing the treatments were withdrawn and released into the CaCl_2_ solution to form the encapsulation [32].

To evaluate the effectiveness of essential oils as natural preservatives in processed foods (sausages), two application methods were used: (1) emulsion (E−); and (2) encapsulation (E+) with alginate [33]. The evaluation was carried out for 30 days with measurements every 10 days.

### 2.4. Chicken Sausage Processing

The chicken sausages were composed of chicken meat (40%), fat (15%), soy protein (13%), starches (7%), salt (3%), sugars and spices (2%), carrageenan (1%) and water at 0 °C (15%). The chicken meat was ground, minced and mixed with the ingredients, and then biopreservatives were applied in liquid form with a micropipette or encapsulation. This was followed by stuffing, tying and heat treatment. (The heat treatment applied during cooking significantly reduces the colony-forming units (CFU) of thermophilic pathogenic microorganisms and completely eliminates the CFU of mesophilic microorganisms, thereby ensuring the microbiological quality and safety of the food product.) After this, the product was cooled, vacuum packed and stored in cold chambers at 0 °C ± 2.

The chicken sausages with the essential oils (treatments), indicated in Table 1, were evaluated in triplicate at 0, 10, 20 and 30 days after storage. To do so, a sample of the product was obtained and sown in Petri dishes with the specific agars for each microorganism, which are detailed in the in vitro phase. The microorganisms evaluated were *E. coli*, *Salmonella*, *Staphylococcus aureus*, mesophilic aerobes and *Clostridium perfingens*. Petri dishes inoculated with the product were incubated at 37 °C for a period ranging from 24 to 36 h. *Clostridium perfingens* was placed in an anaerobic chamber and incubated.

### 2.5. Bromatological Evaluation of Chicken Sausages

The proteins of the chicken sausages were determined using the Kjedahl (Model DKL 20, Shandong, China, 2018) method established in the INEN 1985 standard [34]. To achieve this, 1 g of sample, 15 mL of sulfuric acid, 2 catalyst tablets and 3 anti-effervescent tablets were placed in the digestion tube and the sample was left until it changed colour to black. Subsequently, it was placed in the digester for 3 to 4 h, following the INEN 781 standard [34], at a temperature starting at 100 °C and reaching 400 °C, so that the sample changed to a transparent green colour. It was then placed in the distiller and distilled water and sodium hydroxide were added. In the distiller, the ammonia in the sample was volatilised and collected in boric acid, which contained a mixed indicator, and the colour changed from red to green. Titration was then carried out with 1N hydrochloric acid, where a colour change from green to red was observed. A blank was considered to confirm the results. The amount of nitrogen in the sample was calculated using Equation (1):

(1) $$N\;\left(\%\right)=\frac{0.014\;\left(V_{1}-V_{0}\right)N_{x}}{m}\times 100\;$$

where: *N* = amount of nitrogen, *V*_1_ = volume of HCl consumed in the sample, *V*_0_ = volume consumed by the blank, *m* = weight of the sample and *Nx* = normality of the HCl solution.

The percentage of nitrogen (N) was converted to a percentage of protein by multiplying by 6.25, which is the corresponding factor for a chicken sausage and its chicken derivatives [35] and INEN 1338:2016.

### 2.6. Determination of Fats

For this process, the empty glass balloon was weighed and, once standardised, the chicken sausage sample was crushed and placed in the cellulose cap, which was placed in the SOXHLET (Model Glassco, Madrid, Spain, 2023). Once the structure was assembled, it was heated to 100 °C, so that the solvent (ether) extracted the fat that was retained in the cap. Lipids remained in the collection balloon, so to extract them, a part of the ether was left in the collection balloon and placed in a rotary vapour, which allowed the remaining ether to be extracted. The weight of the glass flask containing the extracted fat was recorded and the fat content was calculated using Equation (2).

(2) $$\;fat\;\left(\%\right)=\frac{P_{2}-P_{1}}{Weight\;sample}\times 100$$

where: *P*_1_ = balloon + fat, and *P*_2_ = balloon on its own.

### 2.7. Moisture Determination (%)

For this, 3.7 g of the chicken sausage sample was used. The BMA I50 Moisture Analyzer (Boeco, Hamburg, Germany) was turned on, the empty aluminium container was placed inside, the sample was positioned and the analyser was closed. After 40 min, the product’s moisture was measured as well as its dry weight to confirm the moisture.

### 2.8. Determination of pH

The pH was determined according to the ISO 2917 reference method [36], i.e., homogenising the sample according to the Laboratory Practice Manual of the Autonomous Metropolitan University of Mexico [37]. The pH reading was taken directly from the Mettler Toledo Seven Compact pH meter (Model Seven Compact Mettler Toledo, Toledo, Spain, 2020).

### 2.9. Sensory Evaluation

The sensory evaluation was carried out according to the NTE INEN-ISO 6658:2014 standard [38], which regulates the general principles of the sensory analysis of food products. The analysis was conducted in the sensory evaluation laboratories of Universidad de Las Américas, with the participation of 30 untrained panellists (blind conditions). The test was conducted in individual booths designed to minimise external stimuli, with white lighting, room temperature (22 ± 2 °C) and ventilation to prevent olfactory interference. To avoid bias, each chicken sausage sample was assigned a code. Data were collected through surveys and the organoleptic parameters were evaluated using a structured 5-point hedonic scale, where 1 means ‘dislike very much’, 2 ‘dislike’, 3 ‘neither like nor dislike’, 4 ‘like a little’ and 5 ‘like very much’.

### 2.10. Statistical Analysis

Prior to the statistical analyses, the Shapiro-Wilks normality test was performed and the variables that did not have a normal distribution were transformed using the square root + 1 transformation. Statistical analyses were performed using the Infostat program (version 2017) and the R package (version R 4.0.3). When statistical differences were present in the ANOVAs for the different sources of variation of interest, Tukey’s test was performed with a statistical significance of 5%.

## 3. Results

### 3.1. In Vitro Evaluation of Essential Oils to Inhibit the Effect of Microorganisms

In vitro, tests evaluated the effectiveness of the essential oils (treatments) against various microorganisms. Statistical differences were observed between treatments for *Clostridium perfringens*, *E. coli* and *Salmonella*, with *p*-values of <0.0001, <0.0001 and <0.0005, respectively, while for *S. aureus*, no statistical differences were found (*p* < 0.0581). For *Clostridium*, treatments T1, T2, T3 and T4 were statistically equal since the DIZ fluctuated between 15.7 and 17.2 mm, presenting a greater effect than the control (0 mm), as shown in Table 2. The same trend was observed for *E. coli*, as the same treatments showed DIZs of 11.3 to 13.1 mm. In Table 2, it is also observed that treatments T1 and T4 restrain *Salmonella* better, as they presented a DIZ of 14.1 mm, while treatments T2, T3 and Te had DIZs of 11.1 to 12 mm.

For *Salmonella*, treatments T1 and T4 presented the highest DIZ (14 mm); thus, the *Salmonella* strains were very sensitive to these treatments. Yet according to the scale proposed by Picazo, *Salmonella* strains were also sensitive to treatments T2 (12 mm), T3 (11 mm) and the control Te (11 mm) [30].

### 3.2. Bromatological Analysis

Before assessing the antimicrobial and sensory effects, a bromatological analysis was conducted on chicken sausages containing encapsulated (E+) and non-encapsulated (E−) essential oils. As shown in Table 3, there were no substantial differences in moisture (57.84% for E+; 58.87% for E−), pH (6.1 for E+; 6.0 for E−), fat (16.70% for E+; 16.80% for E−) or protein content (18.79% for E+; 18.46% for E−). All values complied with the INEN standards for processed chicken sausage.

These results indicate that the incorporation of essential oils—regardless of the application method—did not significantly affect the physicochemical composition of the sausages. Therefore, any variations observed in microbial control or sensory attributes can be attributed to the functional properties of the essential oils rather than to changes in the product’s nutritional or structural matrix.

### 3.3. Evaluation of Essential Oils in Processed Chicken Sausage

#### 3.3.1. *Staphylococcus aureus*

The ways in which the essential oils were applied to the chicken sausages (E− (without encapsulation) and E+ (encapsulated)) were evaluated. When applied to inhibit the growth of *S. aureus*, statistical differences were observed at 0 days (*p* < 0.0459), presenting greater inhibition when the essential oils were applied without encapsulation (0.62 cfu/g) than with it (2.29 cfu/g). In contrast, at 10, 20 and 30 days, no statistical differences were detected in terms of the manner of applying the essential oils (*p* < 0.0628, *p* < 0.2312 and *p* < 0.0625, respectively), as shown in Table 4. It is important to mention that, broadly speaking, the amount of *S. aureus* decreases over time for the 2 ways of applying the essential oils. However, on day 30, there was no presence of the bacteria when applied without encapsulation (E−), while the encapsulated application (E+) caused it to decrease by 50% compared to day 1 (Table 4).

The evaluation of the essential oil treatments applied to chicken sausages at 0, 10, 20 and 30 days revealed no statistically significant differences (*p* < 0.5215, *p* < 0.4433, *p* < 0.2649 and *p* < 0.4841, respectively). In general, a reduction of *S. aureus* was observed in treatments T1, T3 and T4 when comparing day 0 and day 30, but not for T2 and Te, where an increase in cfu/g of bacteria was observed (Table 5).

When evaluating the interaction between the essential oils (treatments) and the E+ and E− forms of application for *S. aureus* at 0, 10, 20 and 30 days, they presented a *p* < 0.4032, *p* < 0.3798, *p* < 0.0270 and *p* < 0.4841, respectively, as shown in Table 6. However, it can be clearly seen that the application of the non-encapsulated essential oils has a greater effect than the encapsulated ones.

#### 3.3.2. *Mesophilic aerobes*

When analysing the efficacy of the essential oils applied to sausages to control mesophilic *A. mesophilus*, highly statistical differences were detected when evaluated at 0, 10, 20 and 30 days since they presented a *p* < 0.0008, *p* < 0.0013, *p* < 0.0078 and *p* < 0.0002. Table 7 shows the bacteria averages when E− and E+ were applied. It is important to point out that there was an initial population of this microorganism in the sausages, which was reduced as time passed by. However, the oils were more effective when applied without encapsulation, as no bacteria were detected by day 30.

Table 8 shows that all treatments, including the control, had the same effect in controlling *A. mesophilus* at 0, 10 and 20 days, as they did not present statistical differences (*p* < 0.8446, *p* < 0.3464 and *p* < 0.7116, respectively). However, on day 30, there were statistical differences (*p* < 0.0019) between the treatments, in that T1, T3, T4 and Te were the best at controlling *A. mesophilus* in the processed chicken sausages.

Regarding the effect of the interaction of the essential oils (treatments) and the form of application (E− and E+) in chicken sausages to control mesophilic *A. mesophilus* (Table 9), it is observed that at days 0, 10 and 20, there are no statistical differences between treatments (*p* < 0.9663, *p <* 0.1093 and *p* < 0.7918, respectively). However, on day 30, significant differences were detected (*p* < 0.0019), with treatment E+T2 showing the highest amount of *A. mesophilus* in the sausages when the encapsulated essential oils were applied.

#### 3.3.3. Coliforms

The reduction of coliforms in the chicken sausages varied depending on the method of essential oil application, as they presented statistical differences at 0, 10, 20 and 30 days (*p* < 0.0069, *p* < 0.0002, *p* < 0.0203 and *p* < 0.0004, respectively). The oils applied without encapsulation performed better than those applied with encapsulation, as shown in Table 10.

The effect of the essential oils used as preservatives to control the presence of coliforms at days 0, 10 and 20 did not present statistical differences (*p* < 0.9750, *p* < 0.7556 and *p* < 0.4681). Therefore, the natural preservatives acted in a similar way to the synthetic preservatives, while on day 30, they presented statistical differences at *p* < 0.0040 (Table 11). Treatment 1 controlled the coliforms (0.28 cfu/g) the best.

The results of the interaction between the way of applying the essential oils (Table 12) showed that there were no significant differences at 0, 10 and 20 days (*p* < 0.4101, *p* < 0.7556 and *p* < 0.6904, respectively). However, on day 30, the interaction did present statistical differences (*p* < 0.0033). The interactions that best-controlled coliforms in the sausages were the essential oils applied without encapsulation, followed by the encapsulated ones, except E+T2, which presented 9 cfu/g.

### 3.4. Sensory Evaluation of Processed Chicken Sausages

In order to find out the effect of essential oils on the sensory characteristics of chicken sausages, colour, taste, smell and texture were evaluated on a hedonic scale from 1 (I do not like it at all) to 7 (I like it very much). A group of untrained people between 18 and 30 years of age was considered. Statistical analyses indicate that there were no differences between the treatments when the oils were applied encapsulated or unencapsulated.

The sensory evaluation averages are presented in Table 13. Overall, the sensory attributes of sausages treated with unencapsulated essential oils were better accepted than those treated with encapsulated oils.

## 4. Discussion

### 4.1. In Vitro Evaluation of Essential Oils

The antimicrobial effectiveness of the essential oils used in this study is associated with the activity of their major phenolic compounds. Carvacrol, found in oregano and thyme essential oils, disrupts the outer and cytoplasmic membranes of *Salmonella* spp. and *Staphylococcus aureus*. Carvacrol also inhibits nucleic acid synthesis and function, induces coagulation of cytoplasmic components and interferes with cell-to-cell communication, leading to complete inhibition [39].

Similarly, eugenol—the principal active component of clove essential oil—exerts its antimicrobial effect by interfering with protein synthesis and metabolic activity in Gram-negative bacteria, disrupting cell function and contributing to bacterial death [31].

These findings align with those of Cáceres et al. [40], who reported that the antimicrobial activity of essential oils is due to the presence of phenolic compounds capable of damaging cell membranes, altering permeability, and compromising the structural integrity of bacterial cells.

### 4.2. Evaluation of Essential Oils in Processed Chicken Sausages

For a food item to be safe and fit for human consumption, it must be free of bacteria. For this reason, synthetic or natural preservatives are intentionally added to food [41]. The synthetic preservative used in this study was one allowed by NTE INEN 1338:2016, while the natural preservatives were the mixture of oregano, thyme and clove essential oils applied in two ways: encapsulated (E+) and directly applied to the chicken emulsion (E-).

From the results obtained for *S. aureus* (Table 5), all chicken sausage products complied with the parameters specified in the NTE INEN 1338:2012 standard [11], as the averages were lower than the 1 × 10^−3^ cfu/g allowed. The effectiveness of oregano and thyme essential oil against *S. aureus* was proven. In the research by Zayed et al. [42], it is mentioned that the microbiological analysis carried out on frankfurters that were smoked and then treated with nisin and clove over a period of 20 days showed an absence of *S. aureus* and *E. coli* (Table 6).

A high mesophilic aerobic count suggests inadequate hygiene, storage and/or handling conditions during food production [43] that do not meet the necessary standards of cleanliness [44]. Food contaminated with mesophilic aerobes can cause illness due to bacterial growth at moderate temperatures. To avoid this contamination, it is necessary for processed foods to include preservatives [43].

The number of bacteria found during our entire study period (Table 8) did not exceed the limits established by NTE INEN 1338:2016 for mesophilic aerobes, which is 1 × 10^5^.

The essential oils applied directly (E−) to the chicken sausage controlled the microorganisms more than twice as much as those applied in encapsulated form (E+), as shown in Table 7. In both cases, it was observed that the amount of mesophilic *A. mesophlus* decreased during the study period. This research indicates that thyme essential oil inhibits the proliferation of these bacteria, as does oregano essential oil [45]. Similarly, Aguilar Paquirachin et al. [22] found that fish fillets coated with oregano essential oil controlled mesophilic aerobes very well at a concentration of 2 × 10^5^ cfu/g.

The presence of coliform species in food is used to evaluate the product’s quality, durability and fitness for human consumption [46]. Our results (Table 12) show that the essential oils applied to the chicken sausages prevented the proliferation of coliforms and the amounts found are within the ranges established by INEN 1338 [11]. In this regard, Vidaković Knežević et al. [47] and Munekata et al. [48] indicate that the application of oregano and thyme essential oil controls *Salmonella*, *Listeria monocytogenes*, *E. coli* and coliforms, possibly due to the presence of secondary metabolites such as carvacrol, thymol, γ-terpinene and p-cymene. This coincides with the results of the ICMSF [46].

Contamination with *E. coli* and *Salmonella* sp. can occur during food production, processing or handling, which can cause serious gastrointestinal illness in humans [46]. Infection generally occurs by consuming contaminated food or water, undercooked ground chicken, unpasteurised milk and raw vegetables [43,46]. The essential oils used in our study allowed an efficient control of these bacteria, as *E. coli* and *Salmonella* were not detected during the period studied, complying with NTE INEN 1338.

Our results agree with those reported by Aldosary et al. [49], who indicate that thyme essential oil controls *E. coli* and *Salmonella*. This is possibly because this plant species has thymol and carvacrol molecules, which act on the cell membrane of Gram-negative bacteria, causing cell death. Oliveira et al. [45], Munekata et al. [48] and Tshabalala et al. [50] confirm the presence of eugenol in clove essential oil, which interferes with the protein synthesis and metabolic activity of Gram-negative microorganisms.

In our research, which evaluated essential oils and nitrating salt, *Clostridium perfringens* bacteria were not detected. Martins et al. [51] investigated the biopreservative power of the combination of thyme, oregano and clove essential oils in mortadella, detecting the anticlostridial action that affects the biofilm and spore formation of *Clostridium perfringens*. Hui [52] investigated the addition of encapsulated oregano and nisin essential oils as an alternative for preserving hamburgers, showing that nisin at concentrations between 2% *p*/*v* and AEO 0.5% *v*/*v* has a high capacity for inhibiting *Clostridium perfringens* and *E. coli*.

### 4.3. Sensory Analysis

From the results obtained in the sensory evaluation of the chicken sausages, it can be concluded that the use of the essential oils of the plant species studied and the synthetic product (nitrating salt) did not affect the organoleptic properties of the product; this agrees with what was indicated by Asghar et al. [53]. It was also found that around 90% of the panellists prefer a product free of synthetic preservatives or artificial ingredients. Overall, the results obtained show a clear consumer trend for products with clean and healthy ingredients on their labels [54].

## 5. Conclusions

The essential oils of oregano, thyme and clove demonstrated antimicrobial effectiveness against *S. aureus*, *E. coli*, *Salmonella* and coliforms in chicken sausage, with all formulations meeting the microbiological criteria established by NTE INEN 1338:2012. These findings support their potential as natural alternatives to synthetic preservatives.

The in vitro results confirmed the antimicrobial activity of these oils at various concentrations, while the in vivo analysis revealed that non-encapsulated applications were more effective than encapsulated ones. Additionally, sausages formulated with non-encapsulated essential oils were more favourably accepted in the sensory evaluation.

In summary, the use of essential oils in chicken sausages represents a safe, effective and consumer-acceptable strategy to reduce the use of synthetic additives and support the development of healthier, microbiologically safe food alternatives.

## Figures and Tables

**Table 1:** Amount of essential oils of the three plant species used in 1 kg of meat product (chicken sausage).

Treatment of Essential Oils	Te	T1	T2	T3	T4
Thyme (μL)		200	200	600	600
Oregano (μL)		50	100	50	100
Clove (μL)		40	40	40	40
Nitrating salt (g)	0.3				

T: treatment.

**Table 2:** Diameter of inhibition zone (DIZ) averages of the in vitro essential oil disc diffusion test on *C. perfringens*, *A. aureus*, *E. coli* and *Salmonella*.

Treatment	*C. perfringens*	S. aureus *	*E. coli*	*Salmonella*
T1	15.7 ± 0.6 b	12.7 ± 0.6	11.7 ± 1.5 b	14 ± 1 b
T2	17 ± 1 b	11.7 ± 0.6	13 ± 1 b	12 ± 0 a
T3	17 ± 2 b	11.7 ± 1	11.3 ± 1.5 b	11 ± 1 a
T4	17 ± 1 b	11.3 ± 0	11.3 ± 0.6 b	14 ± 1 b
Te	0 ± 0 a	11.3 ± 0.6	0 ± 0 a	11 ± 1 a

Note: DIZ values are given in mm. See Table 1 for treatment dosages. * The absence of letters indicates that there are no significant differences between treatments.

**Table 3:** Bromatological parameters of chicken sausages (encapsulated and non-encapsulated) compared with INEN regulatory limits.

Parameter	Encapsulated (E+) *	Non-Encapsulated (E−) *	INEN Min	INEN Max
Moisture (%)	57.84	58.87	-	65
pH	6.1	6.0	-	6.2
Fat (%)	16.70	16.80	-	25
Protein (%)	18.79	18.46	12	-

* The absence of letters indicates that there are no significant differences between treatments.

**Table 4:** Average numbers of *S. aureus* (cfu/g) present in chicken sausages made with different preservatives applied with and without encapsulation.

Form of Application of EO ^1^	0 d	10 d	20 d	30 d
Unencapsulated (E−)	0.62 ± 1.1 b	0.48 ± 1.3 a	0.4 ± 1.1 a	0 ± 0 a
Encapsulated (E+)	2.29 ± 3.1 a	0.96 ± 0.6 a	0.64 ± 0.8 a	1.42 ± 3.3 a

^1^ EO: essential oil; Means followed by the same letters are statistically equal according to Tukey’s test (0.05%).

**Table 5:** Average *S. aureus* (cfu/g) present in chicken sausages with different preservatives.

Treatments	0 d *	10 d *	20 d *	30 d *
T1	2.66 ± 3.4	0.39 ± 0.5	0.67 ± 1	0.17 ± 0.2
T2	1.61 ± 3.6	0.61 ± 1	0.06 ± 0.1	2.11 ± 4.9
T3	1.55 ± 2	0.39 ± 0.5	0.28 ± 0	0.17 ± 0.4
T4	0.94 ± 1.2	1.28 ± 1.1	1.1 ± 1.5	0 ± 0
Te	0.5 ± 0.9	0.93 ± 1.7	0.5 ± 0.8	1.1 ± 2.2

Means followed by the same letter are statistically equal according to Tukey’s test (0.05%). See Table 1 for treatment dosages. * The absence of letters indicates that there are no significant differences between treatments.

**Table 6:** Average number of *S. aureus* (cfu/g) present in chicken sausages produced with different preservatives applied with and without encapsulation.

Interaction Between Form of Application and Essential Oil	0 d *	10 d *	20 d *	30 d *
2E+T1	4.56 ± 3.9	0.78 ± 0.4	1.33 ± 1.2	0.33 ± 0
2E+T2	3.22 ± 5	1.22 ± 1.1	0.11 ± 0.2	4.22 ± 6.7
2E+T3	2.33 ± 2.5	0.78 ± 0.4	0.56 ± 0.4	0.33 ± 0.6
2E+T4	1.11 ± 1.4	1.56 ± 0.2	0.22 ± 0.4	0 ± 0
2E+Te	0.22 ± 0.4	0.44 ± 0.5	1 ± 1	2.22 ± 3
1E−T1	0.77 ± 1.3	0 ± 0	0 ± 0	0 ± 0
1E−T2	0 ± 0	0 ± 0	0 ± 0	0 ± 0
1E−T3	0.77 ± 1.3	0 ± 0	0 ± 0	0 ± 0
1E−T4	0.77 ± 1.3	1 ± 1.7	1.98 ± 1.8	0 ± 0
1E−Te	0.77 ± 1.3	1.41 ± 2.5	0 ± 0	0 ± 0

1E− unencapsulated; 2E+ encapsulated. See Table 1 for treatment dosages. * The absence of letters indicates that there are no significant differences between treatments.

**Table 7:** Averages of *A. mesophilus* (cfu/g) present in chicken sausages with different preservatives applied without and with encapsulation.

Form of Application of EO ^1^	0 d	10 d	20 d	30 d
Unencapsulated (E−)	0.61 ± 1.1 b	0.35 ± 0.9 b	0.46 ± 1 b	0 ± 0 b
Encapsulated (E+)	4.51 ± 3.7 a	1.93 ± 1.9 a	1.98 ± 1.8 a	3.22 ± 5.2 a

^1^ EO: essential oil; means followed by the same letter are statistically equal (Tukey; 0.05%).

**Table 8:** Averages of *A. mesophilus* (cfu/g) present in chicken sausages using different preservatives.

Treatment	0 d *	10 d *	20 d *	30 d
T1	2.66 ± 3.5	1.44 ± 1.9	1.77 ± 2	0.72 ± 1.3 b
T2	3.33 ± 3.7	1.83 ± 2.7	1.22 ± 1.7	5.78 ± 7.5 a
T3	2.69 ± 2.	0.83 ± 0	0.56 ± 0	0.17 ± 0.4 b
T4	1.94 ± 4	1.33 ± 1	1 ± 1.2	0.50 ± 1.2 b
Te	2.16 ± 3.5	0.28 ± 0	1.55 ± 2.	0.89 ± 2.2 b

Means followed by the same letter are statistically equal (Tukey; 0.05%). See Table 1 for treatment dosages. * The absence of letters indicates that there are no significant differences between treatments.

**Table 9:** Averages of mesophilic *A. mesophilus* (cfu/g) present in chicken sausages produced with different preservatives and ways of applying them.

Interaction Between Form of Application and Essential Oils Used	0 d *	10 d *	20 d *	30 d
2E+T1	4.56 ± 4.3	2.89 ± 1.6	2.78 ± 2.3	1.44 ± 1.7 b
2E+T2	5.67 ± 3.8	3.67 ± 2.8	2.44 ± 1.7	11.6 ± 6.4 a
2E+T3	4.89 ± 2.5	0.89 ± 0.5	1.11 ± 0.7	0.33 ± 0.6 b
2E+T4	3.89 ± 5.3	1.67 ± 1.5	1.22 ± 1.3	1 ± 1.7 b
2E+Te	3.56 ± 4.7	0.56 ± 0.4	2.33 ± 3.2	1.78 ± 3.1 b
1E−T1	0.77 ± 1.3	0 ± 0	0.77 ± 1.3	0 ± 0 b
1E−T2	1 ± 1.7	0 ± 0	0 ± 0	0 ± 0 b
1E−T3	0.5 ± 0.9	0.77 ± 1.3	0 ± 0	0 ± 0 b
1E−T4	0 ± 0	1 ± 1.7	0.77 ± 1.3	0 ± 0 b
1E−Te	0.77 ± 1.3	0 ± 0	0.77 ± 1.3	0 ± 0 b

Means followed by the same letter are statistically equal (Tukey; 0.05%). See Table 1 for treatment dosages. * The absence of letters indicates that there are no significant differences between treatments.

**Table 10:** Average coliforms (cfu/g) present in chicken sausages with the application of encapsulated (E+) and non-encapsulated (E−) preservatives.

Form of Applying the Essential Oils	0 d	10 d	20 d	30 d
E−	0.2 ± 0.8 b	0 ± 0 b	0.48 ± 1.9 a	0.1 ± 0.4 b
E+	2.13 ± 2.6 a	2.07 ± 2.1 a	2.04 ± 2.6 a	2.62 ± 3.9 a

Means followed by the same letter are statistically equal (Tukey; 0.05%).

**Table 11:** Average coliforms (cfu/g) in chicken sausages produced with different natural preservatives.

Essential Oil	0 d *	10 d *	20 d *	30 d
T1	1.17 ± 1.7	0.72 ± 0.9	2.43 ± 3	0.28 ± 0.7 b
T2	0.67 ± 1.2	1.39 ± 2	0.61 ± 0.9	4.5 ± 5.5 a
T3	1.22 ± 1.7	0.83 ± 1.4	0.5 ± 0.6	0.33 ± 0.5 b
T4	1.61 ± 3.6	0.56 ± 0.8	0.67 ± 0.9	0.44 ± 0.9 b
Te	1.17 ± 2.4	1.67 ± 3.1	2.11 ± 4.1	1.25 ± 2.4 b

Means followed by the same letter are statistically equal (Tukey; 0.05%). See Table 1 for treatment dosages. * The absence of letters indicates that there are no significant differences between treatments.

**Table 12:** Average coliforms (cfu/g) present in chicken sausages produced with different preservatives and forms of application.

Interaction Between the Application and the Essential Oils	0 d *	10 d *	20 d *	30 d
2E+T1	2.33 ± 1.7	1.44 ± 0.7	2.44 ± 2.4	0.56 ± 1 b
2E+T2	0.33 ± 0.6	2.78 ± 2	1.22 ± 1	9 ± 3.8 a
2E+T3	2.44 ± 1.7	1.67 ± 1.7	1 ± 0.3	0.67 ± 0.6 b
2E+T4	3.22 ± 5	1.11 ± 0.8	1.33 ± 0.9	0.89 ± 1.3 b
2E+Te	2.33 ± 3.2	3.33 ± 4	4.22 ± 5.3	2 ± 3.5 b
1E−T1	0 ± 0	0 ± 0	2.41 ± 4.2	0 ± 0 b
1E−T2	1 ± 1.7	0 ± 0	0 ± 0	0 ± 0 b
1E−T3	0 ± 0	0 ± 0	0 ± 0a	0 ± 0 b
1E−T4	0 ± 0	0 ± 0	0 ± 0	0 ± 0 b
1E−Te	0 ± 0	0 ± 0	0 ± 0	0.5 ± 0.9 b

Means followed by the same letter are statistically equal (Tukey; 0.05%). See Table 1 for treatment dosages. * The absence of letters indicates that there are no significant differences between treatments.

**Table 13:** Organoleptic characteristics for sausages with the application of unencapsulated (E−) and encapsulated (E+) essential oils.

Treatment	Colour		Taste		Smell		Texture	
	E−	E+	E−	E+	E−	E+	E−	E+
Te	4.40 ± 1.35	2.67 ± 0.48	4.97 ± 1.59	3.07 ± 1.23	5.13 ± 1.33	3.83 ± 0.91	4.90 ± 1.69	3.7 ± 0.84
T1	4.80 ± 1.37	4.03 ± 0.93	4.47 ± 1.70	3 ± 1.41	5.13 ± 1.38	3.50 ± 1.11	4.64 ± 1.68	4 ± 0.74
T2	4.67 ± 1.45	4.20 ± 1.10	5.28 ± 1.32	3.13 ± 1.50	5.07 ± 1.28	3.93 ± 0.98	4.87 ± 1.46	3.97 ± 1
T3	4.53 ± 1.53	3.87 ± 1.11	4.83 ± 1.32	4.03 ± 1.10	4.77 ± 1.48	3.97 ± 1.03	4.90 ± 1.40	3.97 ± 0.93
T4	4.38 ± 1.50	3.70 ± 1.21	5.04 ± 1.64	4.47 ± 0.78	4.90 ± 1.54	3.83 ± 0.91	4.53 ± 1.48	4.1 ± 1.03
Average	4.6 ± 0.2	3.7 ± 0.6	4.9 ± 0.3	3.5 ± 0.7	5.0 ± 0.2	3.8 ± 0.2	4.8 ± 0.2	3.9 ± 0.1

T1 = 200 µL of thyme essential oil, 50 µL of oregano essential oil and 40 µL of clove essential oil; T2 = 200 µL of thyme essential oil, 100 µL of oregano essential oil and 40 µL of clove essential oil; T3 = 600 µL of thyme essential oil, 50 µL of oregano essential oil and 40 µL of clove essential oil; T4 = 600 µL of thyme essential oil, 100 µL of oregano essential oil and 40 µL of clove essential oil.

## Data Availability

The original contributions presented in this study are included in the article. Further inquiries can be directed to the corresponding author.

## References

[1] Lisboa H.M., Pasquali M.B., Dos Anjos A.I., Sarinho A.M., De Melo E.D., Andrade R., Batista L., Lima J., Diniz Y., Barros A. (2024). Innovative and Sustainable Food Preservation Techniques: Enhancing Food Quality, Safety, and Environmental Sustainability. Sustainability.

[2] Tubay-Bermúdez C.J., Zambrano L.A., Vera M.M.L., Brigitte K., Jimènez M., Revilla-Escobar K.Y. (2024). Aceites esenciales en la conservación de alimentos: Una revisión. Rev. Científica Técnica Agropecu. Agroindustrial Ambient..

[3] Romero A. (2025). Determinación de Aditivos Alimentarios En Muestras de Alimentos Procesados En Ecuador: Niveles y Riesgos. VITSC.

[4] Schnorr Vargas A.J. (2018). Carnes Procesadas: Un Peligro Para La Salud. OrbTer.

[5] Ma H., Qi X. (2023). Red Meat Consumption and Cancer Risk: A Systematic Analysis of Global Data. Foods.

[6] Lopes A.L., Paulino A.C., Thaumaturgo M.A.S., Araújo W.M., Caloba P., Kawanishi K., Willert K., De Oliveira R.P., Machado J.C., Lemos F. (2025). Dietary Intake of the Red Meat-Derived Glycan Neu5Gc Fuels Colorectal Cancer through up-Regulation of Wnt Signaling Pathway. Cancer Lett..

[7] Díaz M.C., Glaves A. (2020). Relación Entre Consumo de Alimentos Procesados, Ultraprocesados y Riesgo de Cáncer: Una Revisión Sistemática. Rev. Chil. Nutr..

[8] Mendez Criollo M.E., Morocho Pillco C., Espinoza Mejía M., Abril Ulloa V. (2022). Compra y Consumo de Alimentos Después Del Confinamiento Por La Pandemia de COVID-19: Percepciones de Adultos En Cuenca, Ecuador. Cienc. Serv. Salud Nutr..

[9] Carhuallanqui Pérez A., Salazar Salvatierra M.E., Ramos Delgado D. (2020). Efecto Antimicrobiano Del Aceite Esencial de Orégano Frente a Listeria Monocytogenes y Staphylococcus Aureus. Rev. Investig. Altoandin..

[10] Perveen S., Perveen S., Mohammad Al-Taweel A. (2021). Introductory Chapter: Terpenes and Terpenoids. Biochemistry.

[11] (2016). Carne y Productos Cárnicos. Productos Cárnicos Crudos, Productos Cárnicos Curados-Madurados y Productos Cárnicos Precocidos- Cocidos. Requisitos.

[12] Bonfim S.M.V., Leite M.J.S., Camusso I.G., Marchioni D.M.L., Carvalho A.M. (2024). Consumption of Meat in Brazil: A Perspective on Social Inequalities and Food and Nutrition Security. Int. J. Environ. Res. Public Health.

[13] Luna Luna A., Ramírez Chávez G., Manchay Reyes G.J. (2020). Analysis of Poverty in Ecuador, Period 2017–2018. Univ. Soc..

[14] Viteri C., Cabrera J., Iza P., Moreno C., Guanga V. (2024). Consumption of Processed and Ultra-Processed Foods by a Young Population of Ecuador. An Analysis in Light of the PAHO Model. Salud Cienc. Tecnol..

[15] (2022). Estadísticas Económicas.

[16] Rao J., Chen B., McClements D.J. (2019). Improving the Efficacy of Essential Oils as Antimicrobials in Foods: Mechanisms of Action. Annu. Rev. Food Sci. Technol..

[17] Reis D.R., Ambrosi A., Luccio M.D. (2022). Encapsulated Essential Oils: A Perspective in Food Preservation. Future Foods.

[18] Dogruyol H., Mol S., Cosansu S. (2020). Increased Thermal Sensitivity of Listeria Monocytogenes in Sous-Vide Salmon by Oregano Essential Oil and Citric Acid. Food Microbiol..

[19] Masyita A., Mustika Sari R., Dwi Astuti A., Yasir B., Rahma Rumata N., Emran T.B., Nainu F., Simal-Gandara J. (2022). Terpenes and Terpenoids as Main Bioactive Compounds of Essential Oils, Their Roles in Human Health and Potential Application as Natural Food Preservatives. Food Chem. X.

[20] Leyva-López N., Gutiérrez-Grijalva E., Vazquez-Olivo G., Heredia J. (2017). Essential Oils of Oregano: Biological Activity beyond Their Antimicrobial Properties. Molecules.

[21] Gallegos Flores P.I., Valenzuela R.B., Delgadillo Ruiz L., Meza López C., Echavarría Cháirez F. (2019). Actividad Antibacteriana de Cinco Compuestos Terpenoides: Carvacrol, Limoneno, Linalool, α-Terpinemo y Timol. Trop. Subtrop. Agroecosyst.

[22] Aguilar Paquirachín E., Guamuro Fonseca A.M., Minchán-Velayarce H.H., Pasapera-Campos S.E., Ticona Yujra J.A. (2024). Análisis Microbiológico y Sensorial de Filetes de Tilapia (*Oreochromis niloticus*) Con Recubrimiento Bioactivo Incorporando Aceite Esencial de Orégano (*Origanum vulgare*). Pakamuros.

[23] Ozogul Y., Yuvka İ., Ucar Y., Durmus M., Kösker A.R., Öz M., Ozogul F. (2017). Evaluation of Effects of Nanoemulsion Based on Herb Essential Oils (Rosemary, Laurel, Thyme and Sage) on Sensory, Chemical and Microbiological Quality of Rainbow Trout (*Oncorhynchus mykiss*) Fillets during Ice Storage. LWT.

[24] Lages L.Z., Radünz M., Gonçalves B.T., Silva da Rosa R., Fouchy M.V., de Cássia dos Santos da Conceição R., Gularte M.A., Barboza Mendonça C.R., Gandra E.A. (2021). Microbiological and Sensory Evaluation of Meat Sausage Using Thyme (*Thymus vulgaris*, L.) Essential Oil and Powdered Beet Juice (*Beta vulgaris* L., Early Wonder Cultivar). LWT.

[25] Vafania B., Fathi M., Soleimanian-Zad S. (2019). Nanoencapsulation of Thyme Essential Oil in Chitosan-Gelatin Nanofibers by Nozzle-Less Electrospinning and Their Application to Reduce Nitrite in Sausages. Food Bioprod. Process..

[26] Sharma H., Mendiratta S.K., Agrawal R.K., Gurunathan K., Kumar S., Singh T.P. (2017). Use of Various Essential Oils as Bio Preservatives and Their Effect on the Quality of Vacuum Packaged Fresh Chicken Sausages under Frozen Conditions. LWT Food Sci. Technol..

[27] Hudzicki J. (2016). Kirby-Bauer Disk Diffusion Susceptibility Test Protocol.

[28] Yassine I., Rafei R., Pardos De La Gandara M., Osman M., Fabre L., Dabboussi F., Hamze M., Weill F.-X. (2023). Genomic Analysis of Shigella Isolates from Lebanon Reveals Marked Genetic Diversity and Antimicrobial Resistance. Microb. Genom..

[29] Birky C.W., Maughan H. (2020). Evolutionary Genetic Species Detected in Prokaryotes by Applying the K/θ Ratio to DNA Sequences. bioRxiv.

[30] Picazo J. (2000). Métodos Básicos Estudios de La Sensibilidad a Los Antimicrobianos. Procedimientos En Microbiología Clínica.

[31] Li R., Hou G. (2023). Guixiang Hou Biological Activity of Eugenol and Its Latest Progress in Antibacterial Application. Int. Core J. Eng..

[32] Delshadi R., Bahrami A., Assadpour E., Williams L., Jafari S.M. (2021). Nano/Microencapsulated Natural Antimicrobials to Control the Spoilage Microorganisms and Pathogens in Different Food Products. Food Control.

[33] Muchiutti G.S., López Novello L.H., Córsico F.A., Larrosa V.J. (2019). Cápsulas de Alginato Para La Protección de Polifenoles Presentes En El Aceite Esencial de Orégano. Cienc. Docencia Tecnol..

[34] (1985). Carne y Productos Cárnicos. Determinación Del Nitrógeno.

[35] ITW Reagents (2018). Determinación de Nitrógeno Por El Método Kjeldahl.

[36] (1992). Nl. Meat and Meat Products—Measurement of pH (Reference Method).

[37] Cientisol (2023). Medición de pH En Carne y Productos Cárnicos.

[38] (2014). Análisis Sensorial de Alimentos. Metodología. Guía General (ISO 6658:2005, IDT).

[39] Liu Y., Wu L., Han J., Dong P., Luo X., Zhang Y., Zhu L. (2021). Inhibition of Biofilm Formation and Related Gene Expression of Listeria Monocytogenes in Response to Four Natural Antimicrobial Compounds and Sodium Hypochlorite. Front. Microbiol..

[40] Cáceres M.B., Rozo M.F., del Valle García E. (2021). Revista Cientifica FCA.

[41] Enriquez M., Serrano G., Cuadrado D., Ricaurte P. (2024). Efecto de Los Aceites Esenciales de Plantas Aromáticas En La Conservación de Embutidos. Rev. Soc. Cient. Parag..

[42] Zayed H., Mousa M., Youssef H. (2024). Monitoring of Some Food Poisoning Bacteria in Smoked and Cured Meat Products. AJVS.

[43] Al-Mazrouei M.A., Al-Kharousi Z.S., Al-Kharousi J.M., Al-Barashdi H.M. (2024). Microbiological Evaluation of Local and Imported Raw Beef Meat at Retail Sites in Oman with Emphasis on Spoilage and Pathogenic Psychrotrophic Bacteria. Microorganisms.

[44] Rodríguez M., Valero A., Posada-Izquierdo G.D., Carrasco E., Zurera G. (2011). Evaluation of Food Handler Practices and Microbiological Status of Ready-to-Eat Foods in Long-Term Care Facilities in the Andalusia Region of Spain. J. Food Prot..

[45] Oliveira T.S., Almeida R.C.D.C., Silva V.D.L., Ribeiro C.V.D.M., Bezerra L.R., Ferreira Ribeiro C.D. (2025). Enhancing Beef Hamburger Quality: A Comprehensive Review of Quality Parameters, Preservatives, and Nanoencapsulation Technologies of Essential and Edible Oils. Foods.

[46] International Commission on Microbiological Specifications for Foods (ICMSF) (2018). Impact of Sampling Concepts on the Effectiveness of Microbiological Methodologies. Microorganisms in Foods 7.

[47] Vidaković Knežević S., Knežević S., Vranešević J., Kravić S.Ž., Lakićević B., Kocić-Tanackov S., Karabasil N. (2023). Effects of Selected Essential Oils on Listeria Monocytogenes in Biofilms and in a Model Food System. Foods.

[48] Munekata P.E.S., Pateiro M., Rodríguez-Lázaro D., Domínguez R., Zhong J., Lorenzo J.M. (2020). The Role of Essential Oils against Pathogenic Escherichia Coli in Food Products. Microorganisms.

[49] Aldosary S.K., El-Rahman S.N.A., Al-Jameel S.S., Alromihi N.M. (2023). Antioxidant and Antimicrobial Activities of Thymus Vulgaris Essential Oil Contained and Synthesis Thymus (Vulgaris) Silver Nanoparticles. Braz. J. Biol..

[50] Tshabalala R., Kabelinde A., Kaptchouang Tchatchouang C.-D., Ateba C.N., Manganyi M.C. (2021). Effect of Clove (*Syzygium aromaticum*) Spice as Microbial Inhibitor of Resistant Bacteria and Organoleptic Quality of Meat. Saudi J. Biol. Sci..

[51] Martins H.H.D.A., Simões L.A., Isidoro S.R., Nascimento S.D.S., Alcântara J.P., Ramos E.M., Piccoli R.H. (2021). Preservative of Essential Oil Blends: Control of Clostridium Perfringens Type a in Mortadella. Braz. Arch. Biol. Technol..

[52] Hui Y.H. (2012). Handbook of Meat and Meat Processing.

[53] Asghar L., Sahar A., Khan M.I., Shahid M. (2024). Fabrication and Characterization of Chitosan and Gelatin-Based Antimicrobial Films Incorporated with Different Essential Oils. Foods.

[54] Al Bulushi I.M., Al Kharousi Z.S., Rahman M.S., Khan M.S., Shafiur Rahman M. (2021). Vitek: A Platform for a Better Understanding of Microbes. Techniques to Measure Food Safety and Quality.

